# Association of Obesity with Onset of Puberty and Sex Hormones in Chinese Girls: A 4-Year Longitudinal Study

**DOI:** 10.1371/journal.pone.0134656

**Published:** 2015-08-06

**Authors:** Lingling Zhai, Jihong Liu, Jian Zhao, Junxiu Liu, Yinglong Bai, Lihong Jia, Xingjia Yao

**Affiliations:** 1 Department of Maternal and Child Health, School of Public Health, China Medical University, Shenyang, Liaoning Province, China, 110013; 2 Department of Epidemiology and Biostatistics, Arnold School of Public Health, University of South Carolina, Columbia, South Carolina, United States of America 29208; 3 Department of Pharmacology, Shenyang Pharmaceutical University, Shenyang, Liaoning Province, China, 110016; TaipeiCityHospital, TAIWAN

## Abstract

**Objective:**

To examine the influence of childhood obesity on the early onset of puberty and sex hormones in girls.

**Methods:**

Healthy girls with different percentages of body fat at baseline (40 obese, 40 normal, and 40 lean) were recruited from three elementary schools in Shenyang, China. These girls (mean age 8.5 years) were also matched by height, school grade, Tanner stage, and family economic status at baseline. Anthropometry, puberty characteristics, and sex hormone concentrations were measured at baseline and at each follow-up visit. The generalized estimating equation model and analysis of variance for repeated measures using a generalized linear model were used to determine the differences in puberty characteristics and sex hormones among three groups.

**Results:**

Over 4 years, mean age of breast II onset was earlier among obese girls (8.8 years) than normal girls (9.2 years) and lean girls (9.3 years). The prevalence (%) of early-maturation in the obese, normal, and lean groups was 25.9%, 11.1%, and 7.4%, respectively. Obesity was associated with an increased risk for breast stage II (year 2: RR, 6.3; 95% CI, 1.9–21.1 and year 3: RR, 6.9; 95% CI, 0.8–60.1). None of the girls experienced menarche in the first year; however, by the fourth year 50.0% of obese girls had menarche onset, which was higher than normal weight (27.5%) and lean girls (8.1%). The mean estradiol level increased with age in the obese, normal, and lean groups. The mean estradiol concentration was higher in obese girls than in normal and lean girls throughout the 4-year period (*P*<0.05).

**Conclusions:**

Childhood obesity contributes to early onset of puberty and elevated levels of estradiol in girls.

## Introduction

Puberty is a complex temporal sequence of biological events leading to the maturation of secondary sex characteristics, height increase, and attainment of reproductive capacity. The hypothalamus becomes less sensitive to the negative feedback of sex hormones during the onset of puberty. The secretion of gonadotropin releasing hormone (GnRH) from the hypothalamus is increased, and the consequent secretion of follicle-stimulating hormone and luteinizing hormone from the pituitary results in the release of sex hormones from the gonads, and other maturation milestones, such as breast development and menarche.

Since the 19th century, with the overall improvement in public health, the onset of puberty (defined as the age of thelarche) has declined significantly in different populations throughout the world [1–3]. Early onset of puberty in girls is known to have long-term health consequences such as psychological problems, risk-taking behaviors [4], the metabolic syndrome [5], breast cancer [6], and ovarian cancer [7]. Thus, the early onset of puberty in girls is a major public health concern.

Reasons for the early onset of puberty in girls are multiple and include endocrine-disrupting chemicals [8], psychosocial factors (i.e., father absence)[9], and chronic stress [10]. The secular trend of premature thelarche in girls parallels an increasing trend in childhood obesity [2, 3], therefore researchers have started to investigate the association between childhood obesity and early onset of puberty [10, 11].

Previous studies found that body composition was positively associated with younger age at menarche and earlier onset of puberty in obese girls [12–15]. Some studies concluded that obesity is an important contributing factor to the early onset of puberty in girls [16–18]. Furthermore, some studies suggested that girls with early breast development have greater adiposity (body mass index [BMI], skinfold thickness) compared to age-matched girls without thelarche[19].

In China, the most populous country in the world, the age of puberty onset in girls has declined significantly in the past two decades. The age of menarche was 13.3 years in 1985 in urban areas, which was decreased by 0.7 years in 2005 (12.6 years old) and by additional 0.3 years in 2010 (12.4 years old)[20]. Coupled with this trend, the prevalence of obesity increased in China [21,22]. The prevalence of obesity in Chinese girls aged 7–22 years living in urban areas increased from 0.5% in 1985 to 5.0% in 2005 [20]and to 5.6% in 2010 [20].

In spite of these secular trends in childhood obesity and early onset of menarche among Chinese girls, to our knowledge no longitudinal studies have been conducted in China to investigate the influence of obesity on early puberty onset. No longitudinal study reported a relationship between sex hormones, essential factors in the initiation and progression of puberty, and early onset of puberty. Thus, it is warranted to further examine the association between childhood obesity and early puberty onset using the data from a prospective cohort study. We hypothesize that: 1) childhood obesity measured by body fat percent at baseline was associated with the early onset of puberty (measured as the age at thelarche) in girls; and 2) childhood obesity measured by body fat percent at baseline was positively associated with the level of estradiol.

## Data and Methods

### Study design

Data were collected from a cohort of second-grade girls from three elementary schools located in Shenyang, northeast China (n = 120). Obesity was classified according to the percentage of body fatusing skinfold thickness: ≥25% for the obese group, 15%–25% for the normal weight group, and<15% for the lean group [23]. Healthy girls with different body fat percent status at baseline were recruited in 1999: obese (n = 40), normal (n = 40), or lean (n = 40). All included girls were followed for four years until they reached the age of 11 years. We excluded children with a history of heart disease, cerebral trauma, immune disease, or secondary obesity which was mainly caused by a medical condition, such as pituitary disease or an endocrine disorder. Girls in the three groups were matched by height (± 1cm), school grade, Tanner stage, and family economic status at baseline. As these three elementary schools primarily admitted children from well-off families in urban China, we believe that our study sample was homogeneous in terms of socioeconomic status.

During the 4 years, no girls were transferred to different school or dropped out of the school. The follow-up rate was 100% over the four years, although there were some missing data points in some key variables in year 2 (n = 1), year 3 (n = 1), and in year 4 (n = 5).

### Anthropometric measurements

During the 4-year follow-up period, an annual physical examination was carried out by trained doctors who measured the girls’ height, weight, and skinfold thickness using standard procedures. The girls were asked to dress in underwear only and to remove their shoes. Standing height was measured to the nearest 0.1 cm using a stadiometer (TZCS-4, Co., Ltd. Xinman Science and Education Equipment, Shanghai, China). Weight was measured to the nearest 0.1 kg using a leveraged scale (RGT-140, Co., Ltd. Xinman Science and Education Equipment, Shanghai, China). Skinfold thickness was measured on the left side of the body to the nearest 0.1 mm using a Harpenden skinfold caliper (Keman Company, Shanghai, China). Standard methods were used to obtain skinfolds measurements at the four most frequently measured sites (triceps, biceps, subscapular, and suprail). At each site, three measures were taken and the averages of the three were used to calculate body fat percentage.

Body fat percent (BF%) was calculated using the Yao equation [24] on the basis of skinfold data: 7.895967+0.457665x, where x represents the sum of skinfold thickness from the triceps and subscapular area in millimeters. Fat mass was calculated as the product of BF% and body weight. Lean mass was calculated as body weight minus fat mass.

### Measuring puberty onset

At the annual physical examination, the development of secondary sexual characteristics (breast development) was assessed according to Tanner stages by pediatricians who are specialized in child and adolescent health [25]. The onset of menarche was self-reported.

The onset of puberty was defined as the age at which breast stage II occurred [1–3]. In this study, the age of the examination day was used to estimate the age at breast I, breast II and breast III. Early sexual maturation (early onset of puberty)[12] was defined as girls who reached breast stage II stage earlier than the median age for that stage in China (9.20)[26].

### Sex hormone concentrations

Sex hormones were assessed annually using fasting saliva samples (5.0 mL) from each participant [27, 28]. The girls were advised to brush their teeth without toothpaste, to avoid eating, and to thoroughly rinse their mouths with water 30 min before sampling. These samples were collected between 7:00 and 8:00am on the day of follow-up visits and stored at –20°C until testing [29]. The girls who had menses at the time of the visit were advised to provide saliva on menstrual cycle days 6–11.

They were free from contamination (blood, sputum, and water). Testosterone concentrations were measured by radioimmunoassay with DFM-96 type ten-tube radio-immunity and counting apparatus (DPC Company, Beijing, China). The levels of estradiol in saliva were measured using a chemiluminescent enzyme-linked immunoassay according to the manufacturer’s instructions (DPC Company). All samples were assayed in duplicate to minimize system errors. Intra-assay variance was 4.5% for testosterone and 5.1% for estradiol, and between-assay variance was 8.6% and 6.5%, respectively.

### Statistical analysis

Data were presented as means and standard deviations (SD) unless otherwise stated. ANOVA and the least significant difference (LSD) method (pairwise comparison) were used when they were continuous variables. Chi-squared tests were used when the data were categorical variables. We first examined sample baseline characteristics by baseline body fat percent status. Next, we examined the percentage of girls experiencing menarche onset and the age at breast II onset in the three groups. Chi-squared tests and partitions of the X^2^ method were used to compare the percentages of breast development stage in the three groups. Furthermore, generalized estimating equation (GEE) models were used to evaluate the relationship between body fat percentage and breast development (breast stage II). Finally, generalized linear models (GLM) for repeated measures were used to assess mean changes in testosterone and estradiol levels among obese, normal, and lean girls over time and within each group. Significance was set at the 0.05 level. All analyses were performed using SAS version 9.2 (SAS Institute, Cary, NC, USA).

### Consent and ethical approval

Ethics approval was granted by the China Medical University, and the study was performed in accordance with the ethics standards of the committee on human experimentation. We obtain the written agreement of kin, caretakers, or guardians of the girls enrolled in my study. The consent procedure was proved by the Ethics Committee of the China Medical University.

## Results

### Characteristics of the study population

As shown in Table 1, the study sample was recruited from three elementary schools and the baseline body fat percentage status did not differ by school. Their mean age at baseline was 8.5 years of age. Both mean age at baseline and mean height did not differ by the baseline BF%. As expected, the mean weight and BF% were higher in obese girls than in normal and lean girls (*P*<0.05).

**Table 1:** Characteristics of study population at baseline (n = 120, 40/group).

	Obese^a^	Normal^a^	Lean^a^	*P*-value
School ID, n (%) ^b^				0.087
1	20(48.8%)	8(19.5%)	13(31.7%)	
2	12(24.5%)	20(40.8%)	17(34.7%)	
3	8(26.7%)	12(40%)	10(33.3%)	
Age (mean±SD, y)^c^	8.6±0.3	8.6±0.3	8.5±0.4	0.732
Height (mean±SD, cm)^c^	133.2±4.9	132.5±4.9	132.6±5.1	0.761
Weight (mean±SD, kg)^c^	36.9±7.6	28.6±3.0	24.3±2.5	0.000
BF% (mean±SD)^c^	26.4±3.4	17.9±1.5	14.1±0.9	0.000
Age at breast II (95% C.I.)^c^	8.8(8.6–8.9)^d^	9.2(8.9–9.5)	9.3(8.9–9.6)	0.023
Stage of Breast, n (%) ^b^				1.000
I	27(67.5%)	27(67.5%)	27(67.5%)	
II	13(32.5%)	13(32.5%)	13(32.5%)	

^a^. Obese, normal, and lean groups were classified according to body fat percent (%) using skinfold thickness.

^b^. Chi square test of independence were used to compare the difference among the three groups.

^c^. ANOVA and least significant difference (LSD) analyses were used to compare the means among the three groups.

^d^. Post hoc tests were used to compare the difference between obese, normal, and lean girls. The mean age at breast II onset was earlier in obese girls than normal (*P* = 0.04) and lean girls(*P* = 0.02).

During the 4 years, mean height increase was 20.8cm, 20.7cm and 19.4cm for the obese, normal, and lean girl, respectively. But the height increase was not significantly different among the three groups (data not shown). The mean weight of obese, normal weight and lean girls increased by 17.6 kg, 14.4 kg and 11.3 kg, respectively (data not shown).

### Secondary sexual characteristics in girls over the 4-year follow-up period

Two-thirds of the girls experienced stage I breast development and one-third had stage II breast development at baseline, which did not vary by baseline body fat percentage (Table 1). The mean age at breast II was 9.2 years in the normal group; however, the mean age at breast II was 0.5 years earlier in the obese group and 0.1 years later in the lean group (*P* = 0.023) (Table 1).

As shown in Table 2, in the 1^st^ year, the percentages of breast development stage in the three groups were the same. In the 2^nd^ year, we found significant differences in the percentages of girls who were breast II (by Tanner breast staging) between the obese and normal/lean groups; 90.0% in the obese group were breast II compared with 56.4% in the normal group and 50% in the lean group. In the 3^rd^ and 4^th^ year, breast development stages varied in the three groups. In year 3, 37.5% of girls in the obese group and 40% of girls in the normal group were in breast III stage compared with 15.4% of girls in the lean group. In year 4, 74.4% of girls in the obese group and 70% of girls in the normal group were breast III and IV compared with 37.8% of girls in the lean group.

**Table 2:** Stage of breast development in girls during 4 years of follow-up, n*(%).

Group	Year 1		Year 2^a^			Year 3^b^			Year 4^c^			
	I	II	I	II	III	I	II	III	I	II	III	IV
Obese	27(67.5%)	13(32.5%)	4(10.0%)	36(90.0%)	0(0.0%)	1(2.5%)	24(60.0%)	15(37.5%)	0(0.0%)	10(25.6%)	20(51.3%)	9(23.1%)
Normal	27(67.5%)	13(32.5%)	16(41.0%)	22(56.4%)	1(2.6%)	6(15.0%)	18(45.0%)	16(40.0%)	0(0.0%)	12(30.0%)	22(55.0%)	6(15.0%)
Lean	27(67.5%)	13(32.5%)	19(47.5%)	20(50.0%)	1(2.5%)	7(17.9%)	26(66.7%)	6(15.4%)	1(2.7%)	22(5.95%)	13(35.1%)	1(2.7%)

^a^: The percentage of girls who were in different breast stages was significantly different in the three groups (*P* = 0.001, chi-square test). The percentage of obese girls who were in breast stage II and above was significantly higher than those among normal and lean girls (*P* = 0.002, *P* = 0.000, chi-square test).

^b^:The percentage of girls who were in different breast stages was significantly different in the three groups (*P* = 0.028, chi-square test). The percentage of obese girls who were in breast stage II and above was higher than those in lean girls (*P* = 0.023, chi-square test).

^c^:The percentage of girls who were in different breast stages was significantly different in the three groups (*P* = 0.012, chi-square test). The percentage of obese girls who were in breast stage III and above was significantly higher than those in lean girls (*P* = 0.001, chi-square test). The percentage of normal girls who were in breast stage III and above was significantly higher than those in lean girls (*P* = 0.005, chi-square test).

*: Sample sizes for Year 1 were n = 40 girls in each of three groups.

Sample sizes for Year 2 were n = 40 for obese and lean groups, respectively, n = 39 for normal group.

Sample sizes for Year 3 were n = 40 for obese and normal groups, respectively, n = 39 (lean group).

sample sizes for Year 4 were n = 39 (obese), n = 40 (normal) n = 36 (lean).

At baseline, none of the girls experienced the onset of menarche in the first year. By the 4th year, 20 obese girls (50%) had menarche onset, which was higher than the normal girls (n = 11, 27.5%) and lean girls (n = 3, 8.1%) (data not shown).

### Association between obesity and early sexual maturation

The prevalence (%) of early maturation (that is, age at reaching breast stage II earlier than the median age for the stage in the population) was 25.9% in the obese girls, which was higher than that for normal (11.1%) and lean (7.4%) girls, although these differences were not significant statistically (data not shown).

GEE results showed that obesity was a risk factor for breast stage II (b = 1.113, p = 0.0042), and the interaction term between baseline obesity status and follow-up time was significant (b = -1.0777, p = 0.002; data not shown). Due to this significant interaction term, we presented our results by follow-up year in Table 3). Obesity was associated with an increased risk for breast stage II (year 2: relative risk (RR), 6.3; 95% CI, 1.9–21.1] and year 3: RR, 6.9; 95% CI, 0.8–60.1), and the risk of obesity for breast stage II increased from year 2 to year 3 (6.3 to 6.9), although there was no significant difference. Leanness was associated with a reduced risk for breast stage II (year 2: RR, 0.9; 95% CI, 0.4,-2.3 and year 3: RR, 0.8; 95% CI, 0.3–2.7), and the protective role of leanness for breast stage II increased from year 2 to year 3(0.9 to 0.8), although there was no significant difference.

**Table 3:** Relative Risks and 95% Confidence Intervals for being breast II and above during the 4 years.

Covariates		RR(95%CI)		
	Year 1	Year 2	Year 3	Year 4^b^
Obese vs Normal	1.0 (0.4–2.6)	6.3 (1.9–21.1)^a^	6.9(0.8–60.1)	—
Lean vs Normal	1.0 (0.4–2.6)	0.9 (0.4–2.3)	0.8 (0.3–2.7)	—

Abbreviations: CI, confidence interval

^a^: *P*<0.05

^b^: We did not show the 95% CIs of Year 4 due to zero cell in year 4.

### Changes in sex hormone levels

**Fig 1:**
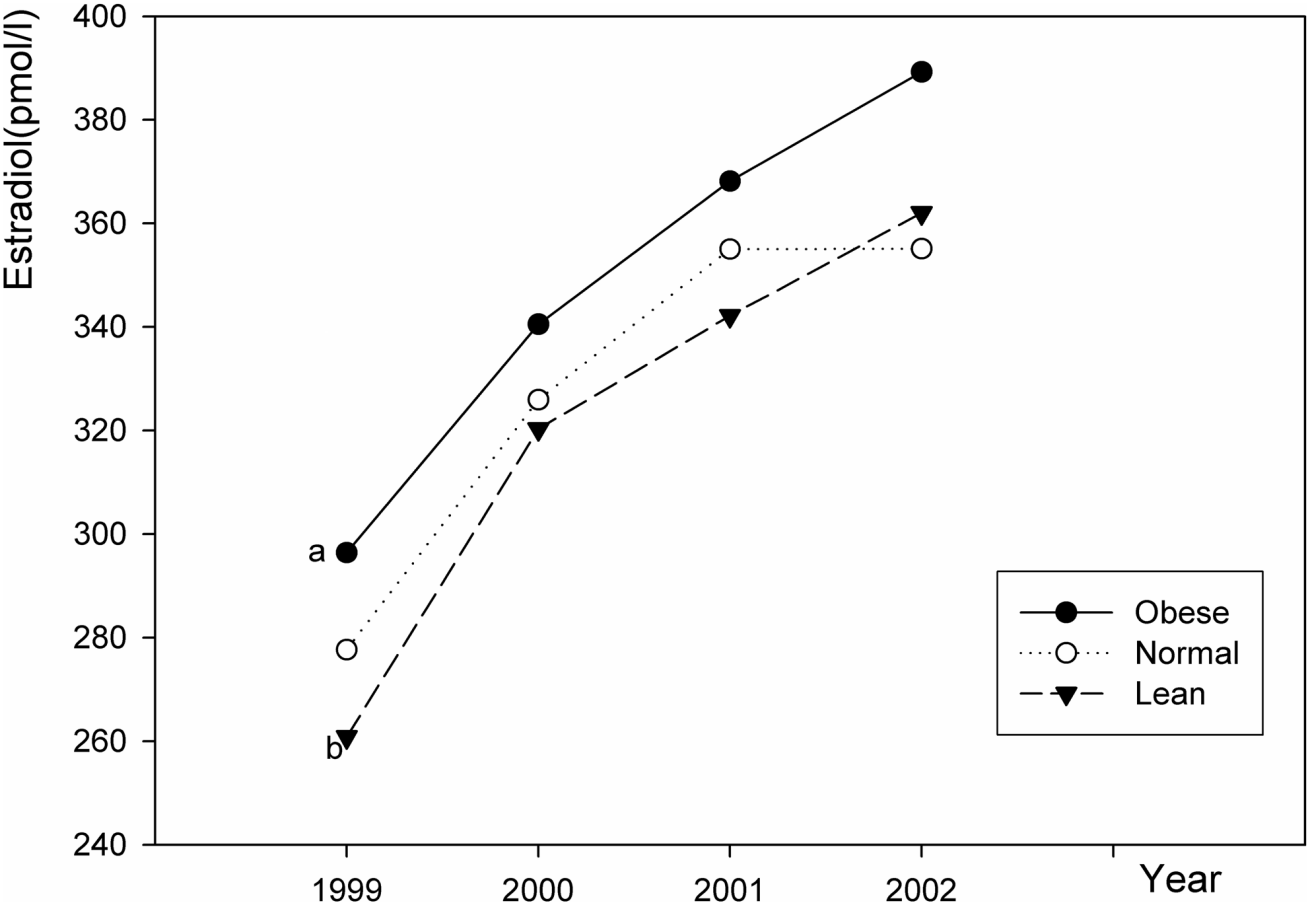
Changes in estradiol levels in girls in the 4 years. a: The trend level of estradiol in obese girls was significantly higher than that in normal weight girls (*P* = 0.043). b: The trend level of estradiol in obese girls was significantly higher than that in lean girls (*P* = 0.003).

**Fig 2:**
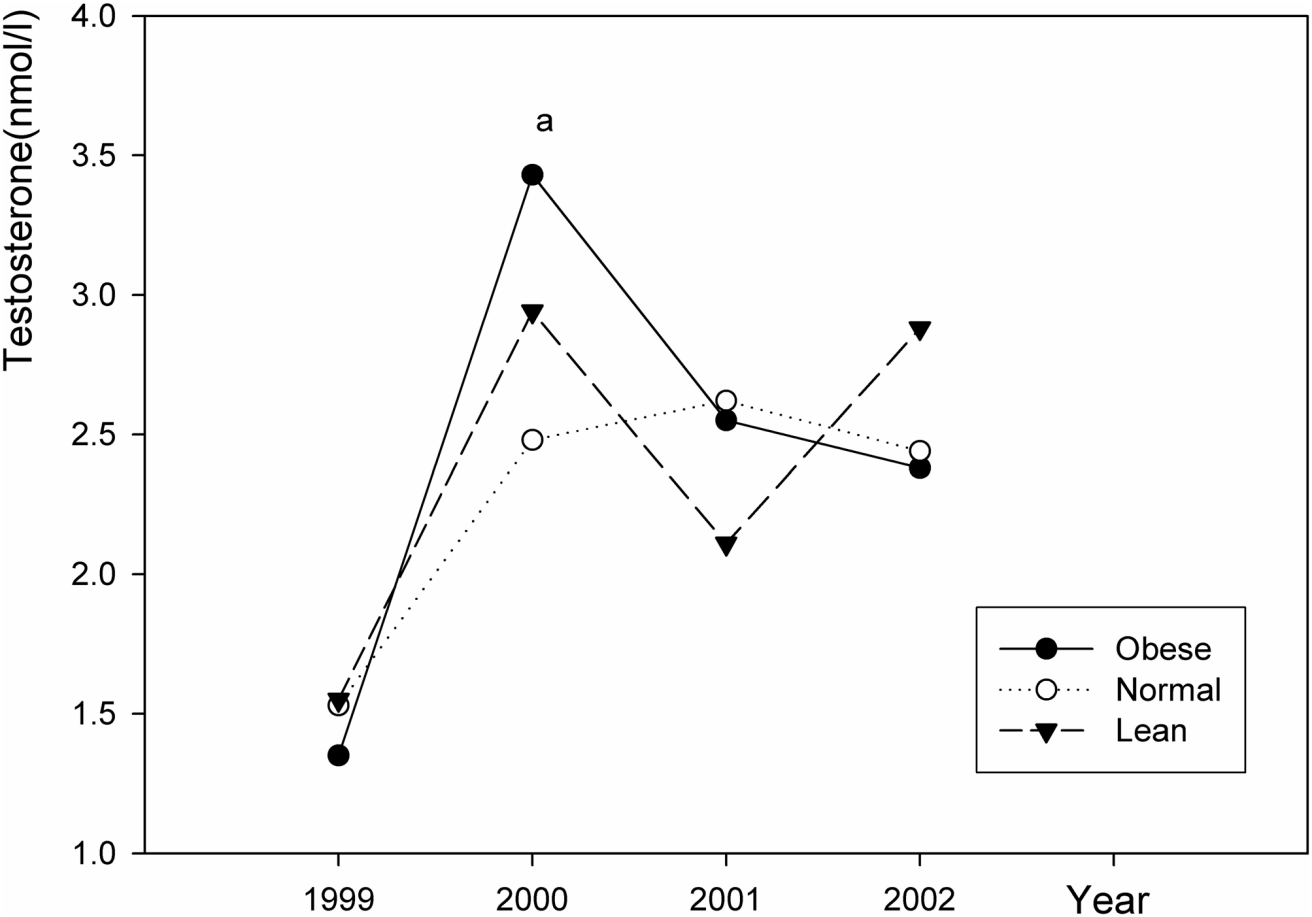
Changes in testosterone levels in girls in the 4 years. a: The testosterone level in obese girls was significantly higher than that in the normal girls (*P* = 0.04).

As shown in Table 4, Figs 1 and 2, the mean estradiol level increased with age in the obese, normal and lean groups. In the 4^th^ year, the mean estradiol level was 389.2, 355.1, and 362.0 pmol/l for the obese, normal, and lean girls, respectively. The mean estradiol concentration was higher in obese girls than in normal and lean girls throughout the four years.

**Table 4:** Generalized Linear Model (GLM) for repeated measures: estradiol and testosterone by baseline group status.

	Source	DF	Type III SS	Mean Square	F value	P-value
Estradiol	Group	2	300621.5	150310.7	20.2	<.0001
	Time	3	2104878.3	701626.1	138.5	<.0001
	Interaction	6	60157.3	10026.2	2.0	0.0657
Testosterone	Group	2	12.9	6.4	0.9	0.4208
	Time	3	591.1	197.0	34.7	<.0001
	Interaction	6	142.9	23.8	4.2	0.0003

The mean testosterone level changed with age in the obese, normal, and lean groups. In the 2^nd^ year, the level of testosterone increased sharply. The mean testosterone level was 3.4 nmol/l in obese girls, which was higher than normal (2.5 nmol/l) and lean girls (2.9 nmol/l). Then, the testosterone level decreased in all three groups. The mean testosterone concentration was not significantly different among the three groups throughout the 4 years.

## Discussion

Most girls experience thelarche (the puberty onset) between 8 and 10 years of age; however, the timing of thelarche may be earlier or later in some girls. In our study, some girls had already undergone thelarche at baseline. Girls in the three groups were matched by breast stage to control for baseline differences in the three groups. Some factors may also influence the time of puberty onset. Some studies have shown that obesity in childhood can result in an earlier onset of puberty in girls [16–18]. In this 4-year prospective cohort study of young adolescent girls, the age at breast II was earlier in obese girls than that for normal girls and earlier than the median age of breast onset in China [26]. The prevalence of early maturation was higher in obese girls compared with normal and lean girls. We also found that obesity was associated with an increased risk for reaching breast stage II. Based on these findings, we concluded that childhood obesity may contribute to the early onset of puberty. Our findings of early onset of puberty in obese girls were consistent with other studies [12–15].

We also found that the risk of obesity for breast stage II increased from year 2 to year 3 (6.26 to 6.88); however, there was no increasing trend for RR (obesity vs. normal) in year 4 because all girls (obese, normal, and lean) were breast stage II when they were old enough. The protective role of leanness for breast stage II increased from year 2 to year 3 (0.91 to 0.81); however, there was no increasing trend for RR (lean vs. normal) in year 4.

The change in sex hormones (such as estradiol and testosterone) is essential to the onset and development of puberty. Abnormal sex hormone level may lead to early puberty or delayed maturation. Increased estradiol production is largely responsible for breast development and changes in the distribution of body fat in pubescent girls. High estradiol levels are associated with precocious (earlier) puberty [2]. Many factors such as endocrine-disrupting chemicals, psychosocial factors, and chronic stress are known to influence the level of estradiol [2]. In this study, the mean estradiol concentration was higher in obese girls than in normal and lean girls suggesting that childhood obesity is associated with elevated levels of estradiol in girls. Other studies also showed that higher levels of estradiol may lead to earlier thelarche and pubertal progression [15,30], indicating that high estradiol levels are associated with early breast development in obese girls.

Body fat stores might influence estradiol level through a few underlying mechanisms. First, adipose tissue has aromatase action and obesity may result in higher peripheral conversion of androstenedione to estrone and testosterone to estradiol [30]. Second, adipose tissue is also related to increased insulin resistance, which lowers sex hormone binding globulin levels leading to increased bioavailability of sex steroids [2]. Finally, long-term obesity can also result in decreased hepatic inactivation by estrogen-2-hydroxylation which leads to reduced estrogen clearance and a corresponding increase in estradiol blood levels [31].

In addition to estradiol, we also examined the levels of testosterone in the three groups. We found a small elevation in testosterone was found in obese girls in the second year. Yet overall we did not find a significant difference in testosterone levels between obese, normal, or lean girls. This finding was consistent with previous studies that not all obese girls have hyperandrogenemia [31,32], and obese girls with early onset of puberty had normal androgen concentrations [33,34]. In contrast, other studies found that peripubertal obesity in girls is associated with hyperandrogenemia [31,34]. Thus, future studies should be performed to examine the relationship between peripubertal obesity and androgens in girls.

This study has a few limitations. First, the study was conducted in Shenyang, China and the results may not be applicable in other geographical areas of the world. However, we believe the biological mechanism underlying obesity and early onset of puberty should be the same in different populations. Second, the study included a small sample size. However, we chose three groups with different body weight status who were matched by several confounding factors, and other studies with a similar sample size also showed significant effects [35]. Third, in our analysis, we used the baseline body fat percentage as the independent variable and did not change the group status in our analysis. This might be limited because four obese girls at baseline changed to normal weight at the end of this study and two more girls who were in the normal group at baseline were shifted to obese group at the end of this study. When we deleted those six girls (<5% of the total) from our analyses, the results were unchanged. Forth, there might be some measurement error in evaluating Tanner stage in obese girls using visual inspection of breast method. Given that trained physicians did all assessment in this study, we considered measurement error of Tanner stages might be minimal in this study.

The BF% based on skinfold caliper measurement was used in this study. Measurement of skinfold thickness is suitable for assessment in large numbers of children and adolescents and has good feasibility, low cost, no side effects, and reasonable accuracy [36]. Oeffinger [37] concluded that accurate measures of BF% can be obtained using skinfold measurement. But it is important to use equations (on the basis of skinfold data) which are best suited for a specific population [37]. Our team previously reported that the skinfold thickness of the humerus triceps and the inferior angle of the scapula, separately or combined, was representative of subcutaneous fat within the trunk, extremities, and entire body, and also developed the equations (Yao equation) on the basis of skinfold data suited for girls 8–12 years of age [24]. Yao’s equation and using the cut-off point (body fat > 25%) as obesity had been used widely in Chinese study and was proved suitable in Chinese girls (8–12 years old)[38,39]. While our results might not be directly comparable with other studies, we think it is important to emphasize the internal validity.

Salivary sex hormone levels were correlated positively with those in blood [27]. Sex steroids, including testosterone and estradiol, have been analyzed successfully in saliva for years [28]. Salivary sex hormone measurements can be influenced by sample collection methods and storage conditions. Strict protocols for collection and storage procedures were carried out in this study.

Considering that early onset of puberty is associated with a myriad of long-term health consequences and obesity itself also results in various adverse health outcomes [4–7], our finding that childhood obesity contributes to the early onset of puberty in girls suggest a strong need for programs and interventions among school-aged children to help them maintain a healthy weight. In terms of research, our study provides data for the design of future studies, which should be larger and longitudinal in terms of study design and include various biomarkers to identify the potential mechanism underlying the association between childhood obesity and the early onset of puberty in girls.

## Conclusions

We found that girls who were obese at baseline were more likely to experience earlier thelarche as evidenced by the younger age of breast II onset in obese girls compared to normal and lean girls and the significantly higher risk of reaching breast II and above in the obese girls compared with the normal girls in year 2. In addition, mean estradiol concentration was higher in obese girls than in normal and lean girls. We conclude that childhood obesity contributes to early onset of puberty and elevated levels of estradiol in girls.

## Supporting Information

S1 File Data. This is the data of the paper.
(SAS)

S2 File legend-data. This is the legend of variable in the paper.
(PDF)
