# Supplementary material for: Association of Obesity with Onset of Puberty and Sex Hormones in Chinese Girls: A 4-Year Longitudinal Study
Source: PLoS One. 2015 Aug 6;10(8):e0134656. doi: 10.1371/journal.pone.0134656 (PMC4527677; doi:10.1371/journal.pone.0134656)
Supplement: S2 File — This is the legend of variable in the paper. (PDF) [file pone.0134656.s002.pdf]

Variable name	legend
number	Personal number
school1	1-schoo1<br>2-school2<br>3-school3
group	1-obesity<br>2-normal<br>3-lean
AGE	Age in 1999
Testos1-4	testos1-testosterone level in 1999<br>testos2-testosterone level in 2000<br>testos3- testosterone level in 2001<br>testos4-testosterone level in 2002<br>nmol/l
Estro1-4	Estro1-estradiol level in 1999<br>Estro1-estradio level in 2000<br>Estro1-estradio level in 2001<br>Estro1-estradio level in 2002<br>pmol/l
Height1-4	Height1-Height in 1999<br>Height2-Height in 2000<br>Height3- Height in 2001<br>Height4-Height in 2002
Weight1-4	Weight1-Weight level in 1999<br>Weight2-Weight level in 2000<br>Weight3-Weight level in 2001<br>Weight4-Weight level in 2002
MENSES1-4	MENSES1-menstruation in 1999<br>testos2-menstruation in 2000<br>testos3- menstruation in 2001<br>testos4-menstruation in 2002<br>1-yes<br>0-no
BREAST1-4	BREAST1-breast development in 1999<br>BREAST2-breast development in 2000<br>BREAST3-breast development in 2001<br>BREAST4-breast development in 2002<br>1-Tanner level1<br>2-Tanner level2<br>3-Tanner level3<br>4-Tanner level4
